# Improving Communication Between the Community Learning Disability Team, Patients, Their Carers and Primary Care Services Within Aneurin Bevan University Health Board

**DOI:** 10.1192/bjo.2024.242

**Published:** 2024-08-01

**Authors:** Kathleen Scanlon, Ceri Richings, Gareth Howe

**Affiliations:** Aneurin Bevan University Health Board, South East Wales, United Kingdom

## Abstract

**Aims:**

Recommendations from the NHS and the Royal College of Psychiatrists advise that patients receive a copy of all correspondence that is sent on to the GP. Often, within psychiatric services, letters are not routinely sent to patients.

To improve communication with patients and their carers, the Aneurin Bevan Learning Disability team have been writing letters directly to patients and sending a copy to the GP.

There is limited use of jargon, and the complexity of the language aims to reflect the individual's reading level.

This study aimed to gain feedback from local GPs on the new letter format to ensure effective communication between teams.

**Methods:**

We contacted Primary Care Services in the local area to gain feedback on how our clinic letters were being processed, we were informed that letters are reviewed by an admin team and only sent on to GPs if there are specific tasks to action, meaning that many of our letters remain unread.

We identified 16 GP practices in the Torfaen and Monmouthshire area and sent a survey by email to gain feedback on the new correspondence style. The survey was sent out three times within a 12-month period.

**Results:**

Of 16 GP practices just 6 responded, with just 1 GP stating that they had noticed a change in the letter style. 50% of GPs felt they received relevant information in the letters from the CLDT. The comments were largely positive with suggestions reflecting changes that have already been made. There is no feedback that suggests GPs feel they are not receiving adequate clinical information.

**Conclusion:**

The lack of response may highlight how infrequently GPs are reviewing the letters from the CLDT confirming the importance of prioritising doctor–patient correspondence. The limited communication from GP to CLDT emphasises the need for improvement in liaison between secondary and primary care services.

The lack of negative feedback about the letters is encouraging. There is no feedback that suggests GPs feel they are not receiving adequate clinical information and clear feedback that GPs want clear and accessible information, particularly regarding specific actions for GPs.

A clear limitation of this work is the lack of response to our survey. Reviewing these letters from our team is a very small proportion of a GPs workload, more time may be needed to ensure GPs have had contact with our team and are able to provide more detailed feedback.

